# Long non‐coding RNA POU6F2‐AS2 promotes cell proliferation and drug resistance in colon cancer by regulating miR‐377/BRD4

**DOI:** 10.1111/jcmm.15070

**Published:** 2020-02-26

**Authors:** Guangru Xu, Hongxing Zhu, Jinhua Xu, Yan Wang, Yang Zhang, Minghui Zhang, Dichao Zhu

**Affiliations:** ^1^ Department of Oncology People's Hospital of Pudong Shanghai China; ^2^ Shanghai University of Medicine&Health Sciences Shanghai China

**Keywords:** cell proliferation, colon cancer, drug resistance, LncRNA POU6F2‐AS2, miR‐377/BRD4

## Abstract

The aim of this study was to explore the molecular mechanism of lncRNA POU6F2‐AS2 in proliferation and drug resistance of colon cancer. Total paired 70 colon cancer and adjacent normal tissues were collected from colon cancer patients. Colon cancer and normal colonic epithelial cells were purchased. POU6F2‐AS2 was up‐ or down‐expressed by vectors. LC50 of all cell lines before and after transfection with these plasmids was detected. qRT‐PCR was used to detect the expression of POU6F2‐AS2, miR‐377 and BRD4 before or after transfection. In situ hybridization was also undertaken to detect the level of POU6F2‐AS2. Different concentrations of 5‐Fu (0, 1, 2.5, 5, 10, 20, 40 and 80 μg/mL) were used for 5‐FU insensitivity assay. CCK‐8 and crystal violet staining assay were used for detecting cell proliferation, and flow cytometry was used for identifying cell cycle distribution and apoptosis. In order to detect the fragmented DNA in apoptotic cells, TUNEL assay was used. RNA pull‐down assay and luciferase reporter assay were used to verify the binding site. Rescue assay confirmed the subtractive effect of miR‐377 inhibitors. POU6F2‐AS2 was highly expressed in colon cancer, which was associated with clinical pathology. Up‐regulated POU6F2‐AS2 promoted cell proliferation and cell cycle of colon cancer cells. Overexpression of POU6F2‐AS2 inhibited the expression of miR‐377 and then up‐regulated the expression of BRD4. Up‐regulated BRD4 ultimately promoted cell proliferation and cell survival Down‐regulated POU6F2‐AS2 showed enhanced sensitivity of 5‐FU. POU6F2‐AS2 promoted cell proliferation and drug resistance in colon cancer by regulating miR‐377/BRD4 gene.

**Highlights**

1. LncRNA POU6F2‐AS2 is highly expressed in colon cancer, which is associated with clinical pathology.
2. Overexpression of lncRNA POU6F2‐AS2 promotes cell proliferation and cell cycle of colon cancer cells.
3. POU6F2‐AS2 inhibits the expression of miR‐377 and then up‐regulates the BRD4 expression.
4. Up‐regulated BRD4 ultimately promotes cell proliferation and cycle.
5. Higher expressed lncRNA POU6F2‐AS2 leads to 5‐FU insensitivity.

## 1 INTRODUCTION

Colon cancer is the third gastrointestinal tumour that occurs at the junction of the rectum and the sigmoid colon.^1^ The incidence of colon cancer is increased in people aged 40‐50, and the ratio is 2 to 3:1 for male: female.^2^ In the middle and late stage, patients are always with characters of abdominal distension, indigestion, abdominal pain, mucus or sticky stool.^3^ Companying with tumour ulceration, blood loss and toxin absorption, patients show more serious symptoms, including anaemia, hypothermia, fatigue, weight loss and lower extremity oedema.^4^ Colon cancer in early stage can be cured by endoscopic minimally invasive treatment, while for middle or late stage, the main treatment method is surgery.^5^ Chemotherapy, immunotherapy, traditional Chinese medicine and other supportive treatments are supplement to improve the surgical resection rate, reduce the recurrence rate and improve the survival rate.^6^ However, colon cancer is difficult to diagnose in early stage.^7^ Thereby, more obviously molecular mechanisms of colon cancer are urgently needed for improvement and treatment for patients.

Recent study confirmed that various long non‐coding RNAs (lncRNAs) regulated pathological and physiological process of colon cancer.^8^, ^9^, ^10^ LncRNA POU6F2‐AS2 was a novel biomarker which affected the occurrence and development of oesophageal cancer.^11^ In addition, it could interact with Ybx1 and further participate in development of oesophageal squamous cell carcinoma.^12^ The molecular mechanism of lncRNA POU6F2‐AS2, regulated genes and pathways in colon cancer was unclear so far. Interestingly, various studies confirmed that miR‐377 couldrestore phenotype of cancer cell or make cell line sensitive to cisplatin. For example, Azizi et al^13^ reported that miR‐377 could suppress the expression of DNMT1 and demethylate tumour suppressor genes, and further reverse cancerous phenotypes of pancreatic cells. Besides, miR‐377 was also confirmed to inhibit proliferation and invasion of cervical cancer cells by regulating the expression of Zinc Finger E‐box‐Binding Homeobox 2.^14^ In gastric cancer, it was also found to repress the expression of VEGFA and then suppress proliferation and metastasis of cells.^15^ Besides, BRD4 was confirmed to be frequently down‐regulated by aberrant promoter hypermethylation in human colon cancer in previous study.^16^ BRD was found to be negatively related to miR‐377 in our pre‐experiment. However, the target relationship between BRD and miR‐377 has not been reported clearly. In‐depth study of these lncRNA, miRNA and genes was important for the prevention and treatment of colon cancer.

In this study, we found that lncRNA POU6F2‐AS2 was highly expressed in colon cancer, and POU6F2‐AS2 was also associated with clinical pathology. The aim of this study was to explore the molecular mechanism of lncRNA POU6F2‐AS2, such as proliferation and drug resistance in colon cancer, which might provide a new target for treatment of colon cancer.

## 2 MATERIALS AND METHODS

### 2.1 Tissue samples obtain and cell lines culture

A total of paired 70 colon cancer and adjacent normal tissues were collected from colon cancer patients who undergoing laparoscope surgery in People's Hospital of Pudong from Jan 2012 to Mar 2013. The study was approved by the ethics committee of the People's Hospital of Pudong. All patients or their families signed the informed consent. The follow‐up time was 5 years. After the surgery, the tissues were frozen immediately in liquid nitrogen and stored at −80°C.

Colon cancer cell lines, including HT‐29, HCT‐116, SW620 and OUMS23, and normal colonic epithelial cells (NCM460) were purchased from American Type Culture Collection (ATCC). All cell lines were cultured in DMEM medium with 10% foetal bovine serum (FBS) at 37°C in incubator with 5% CO_2_. All cell culture reagents were from Invitrogen‐Life Technologies.

### 2.2 Transfection and drug treatment

miR‐377 mimics or inhibitor, short hairpin RNA (shRNA) which targeted lncRNA POU6F2‐AS2 and relative controls were obtained from GenePharma Co., Ltd. To overregulate or down‐regulate, the expression of lncRNA POU6F2‐AS2, HT‐29 and SW620 cells was transfected with lncRNA POU6F2‐AS2 (pBabe‐puro‐POU6F2‐AS2) or sh‐POU6F2‐AS2 (pLKO.1‐POU6F2‐AS2) using Lipofectamine^®^ 2000 (Invitrogen‐Life Technologies), respectively. The concentration of DNA for transfection was 300 μg/mL. After transfection for 24 hours, the virus was collected and infected with the prepared HT‐29 and SW620 cells for 24 hours. Fresh virus was added and re‐infected for 24 hours. At this time, the cell density is about 60%. The cells were re‐plated to a density of 25%, and 6 μg/μL of puromycin was added for screening. After 7 days, the puromycin concentration was 3 μg/μL, and the screening was continued for 7 days to obtain a stable transfection system. The expression of lncRNA POU6F2‐AS2 was detected by reverse transcription‐quantitative polymerase chain reaction (RT‐qPCR, Kapa Biosystems). After constructing over‐ or low‐expression stable cell line of HT‐29, the cells were treated with different concentrations of 5‐Fu (0, 1, 2.5, 5, 10, 20, 40 and 80 μg/mL, Shanghai Fortuneibo‐tech Co., Ltd). Empty vector was transfected cell lines as controls.

### 2.3 In situ hybridization (ISH)

LncRNA POU6F2‐AS2 ISH staining was undertaken based on operation manual of ISH kit (Guangzhou Boye Biological technology Co., Ltd).^17^ In short, the sample was fixed and embedded in paraffin, then incubated in graded alcohols and then incubated 30 minutes with 3% hydrogen peroxide. Importantly, the streptavidin‐horseradish peroxidase conjugate and biotin‐conjugated probes were added in samples for hybridization. Finally, haematoxlin was used to stain the sample, and a light microscope (Leica) was applied for observing.

### 2.4 RNA isolation and qRT‐PCR

For total RNA isolation isolation, Trizol reagent (Thermo Fisher Scientific, Inc) was added to cells or prepared tissues according to the operation manual, and then, the obtained RNA was quantized by UV spectrophotometer (U‐3000 Spectrophotometer, Hitachi). TaqMan MicroRNA Reverse Transcription kit, TaqMan High‑Capacity cDNA Reverse Transcription Kitand TaqMan PCR Master Mix (Applied Biosystems) were used for miRNA and mRNA analysis based on the manufacturer's instruction. A final 20 μL reaction with samples, reverse transcriptase, polymerase and tagged oligo (dT) primers was used for cDNA synthesis. The 2^−ΔΔCq^ method was applied to detect the relative expression levels as previously described,^18^ and the PCR conditions were as follows: 1 cycle of 95°C for 30 seconds; 40 cycles of 95°C for 5 seconds and 60°C for 30 seconds; one cycle of 95°C for 5 seconds, 60°C for 1 minutes and 95ºC for 15 second. β‐actin was used as reference. The sequences of primer for q RT‐PCR were shown in Table 1.

**Table 1:** The sequences of primer for q RT‐PCR

	Up‐stream	Down‐stream
lncRNA POU6F2‐AS2	ACAGCAGTGCCAGAAGGAGT	TTTGCAGACCTGAGCTTGTG
BRD4	CCACACTGCGTGAGCTGGAG	ATCTTGGAGGAGCCGGCAAT
β‐actin	TCCCTGGAGAAGAGCTACGA	AGCACTGTGTTGGCGTACAG
miR‐377	ATCACACAAAGGCAAC	GTGCAGGGTCCGAGGT
U6	CTCGCTTCGGCAGCAGCACA	AACGCTTCACGAATTTGCGT

### 2.5 In vivo xenograft model

The animal experiments were approved by Biomedical Ethics Committee of People's Hospital of Pudong. The transfected stable HT‐29 cell line with lower expression of lncRNA POU6F2‐AS2 was subcutaneously injected into nude mouse, and HT‐29 cells transfected by same vector with scrambled sequence were also injected as control. The tumour volume was measured. When the tumour volume reached 300 mm^3^, 5‐FU (100 mg/kg) was intravenously via a catheter placed into the lateral tail vein of xenograft mouse. The xenograft models were divided into four groups, including pLKO.1+Vehicle group, pLKO.1+5‐FU group, pLKO.1‐POU6F2‐AS2 +Vehicle group and pLKO.1‐POU6F2‐AS2 +5‐FU group. The tumour volume was measured at 35th day after 5‐FU or vehicle treatment.

### 2.6 TUNEL and Ki67 staining in colon cancer cell lines

In order to detect the fragmented DNA in apoptotic cells, the in situ cell death detection kit (POD, Roche) was used for TUNEL assay. The cells were dipped twice with xylene for 5 minutes each time, washed once with gradient ethanol and treated by cell permeate for 8 minutes. The treatment group was mixed with 50 μL of TdT+450 μL of fluorescein‐labelled dUTP solution. After the slides were dried, 50 μL of TUNEL reaction mixture was added to the specimen. Apoptotic cells were counted. A total of 50 μL converter‐POD and 50‐100 μL DAB substrate were added. After taking a photograph, the slide was counterstained with haematoxylin and rinsed with tap water immediately. Then, the sample was treated with gradient alcohol dehydration, xylene transparent and neutral gum seal. A drop of PBS was added to the field of view, and cells were counted.

Simultaneously, Ki67 staining kit (Zhongshang Jinqiao Biotechnology Co., Ltd.) was used to detect the growth index of colon cancer tumour cells.^19^ Detail processes were followed as the manufacturer's instruction. In short, the sample was deparaffnized and rehydrated, and 10 mmol L^–1^ citrate buffer was used for antigen retrieval. After the activity of endogenous peroxidase was blocked, primary antibody and anti‑Ki67 monoclonal antibody were added to incubate. Positive cells of per microscopic field were counted.

### 2.7 Colony formations

After transfection, the cells were digested with 0.25% trypsin, and the cells were suspended in 10% foetal bovine serum in RPMI 1640 medium for use. The cell suspension was diluted as a gradient and seeded in a Petri dish with an appropriate cell density. The cells were allowed to stay for 2 weeks in an environment of 37°C with 5% CO_2_ and saturated humidity. The supernatant was discarded and carefully immersed twice with PBS. Then, the fixative solution was removed, an appropriate amount of GIEMSA application staining solution was added and stayed for 10‐30 minutes, and then, the staining solution was slowly washed away with running water. The number of clones greater than 10 cells was counted under a microscope. Finally, the clone formation rate was calculated.

### 2.8 Proliferation assays and cell viability analysis

The cell proliferation and cell viability were detected by cell counting kit‐8 (CCK8, GLPBIO). Briefly, after knockdown or overexpression of lncRNA POU6F2‐AS2, the cells were seeded in 24‐well plates with density of 1 × 10^4^. After culture for 24, 48 and 72 hours, 10 µL CCK8 was added to each well and OD450 was detected bymicroplate reader (BioTek Instruments). After 5‐Fu treatment for 48 hours, the cell viability was also detected by CCK8 assay.

### 2.9 Cell cycle analysis and apoptosis assays

The transfected cells were seeded in 6‐cm dish with density of 1 × 10^5^. The medium was changed once per day. After culture for 72 hours, 75% cold ethanol was used to fix and permeabilize the cells. After PBS washing, propidium iodide buffer was added to resuspend these cells. Flow cytometry was processed for detecting cell cycle. For apoptosis assays, fluorescein isothiocyanate‐conjugated annexin V and propidium iodide were applied for staining, and flow cytometry(FACScan, Beckton Dickinson) was used to analyse the percentage of apoptotic cells. FL2 channel was used in this experiment.

### 2.10 Binding site prediction and RNA pull‐down assay

We processed a catRAPID analysis and obtained the predicated binding site of lncRNA POU6F2‐AS2. RNA pull‐down assay was processed in SW620 cell line with up‐regulated lncRNA POU6F2‐AS2. 5‐bromo‐UTP (BrU) labelled POU6F2 sense RNA, antisense RNA and control were synthesized. Based on the manufacturer's instruction, the experiment was processed by the Pierce™ Magnetic RNA‐Protein Pull‐Down Kit (Thermo Fisher Scientific). The labelled RNAs were combined with anti‐BrU antibody‐conjugated magnetic protein A/G beads. Extracted cytoplasmic was incubated with the RNA/antibody/beads mixture for 2 hours. Then, the beads were washed by buffer II. Unspecific bound proteins were removed by repetitive washing with buffer D containing 4 mmol L^‐1^ magnesium chloride. RNA/protein complexes were eluted, resolved by gel electrophoresis followed by staining with the SilverQuest™ Silver Staining Kit (Life Technologies) and detected using mass spectrometry analysis. Proteins only specific binding to lncRNA POU6F2‐AS2 sense RNA were ranked according to spectra counts.

### 2.11 Western blot analysis

The expressions of BRD4 and cancer resistance‐related gene (P‐gp, MRP2 and BRCA2) were detected by Western blot analysis. Colon cancer cells were lysed, and total protein was extracted and determined by Bradford method. Different molecular weight proteins were separated by sodium dodecyl sulphate‐polyacrylamide gel electrophoresis (SDS‐PAGE). The protein was transferred to a nitrocellulose membrane, stained with Coomassie brilliant blue and eluted. The membrane was blocked overnight with TBS buffer containing 5% skim milk powder. The goat anti‐rat primary antibody (1:150, Sigma) was added for hybridization, then horseradish peroxidase‐labelled rabbit anti‐goat IgG secondary antibody (1:500, Sigma) was used for immunoreaction, and DAB was developed. The results were analysed in an image analysis system (Scion Corporation).

### 2.12 LC50 assay

The cells in the logarithmic growth phase were inoculated into the 96‐well plate at 1 × 10^4^/mL. After 14 hours of culture, the cells were completely adhered and the culture solution was discarded. Different concentrations of transfection solution were added and cultured for 48 hours. Total 20 μl 5 mg/mL MTT was added to each well, and after 4 hours, the supernatant was discarded and DMSO was added. After fully dissolving the crystal, the absorbance value was measured by a microplate reader (BioTek Instruments). The LC50 at each concentration of the cells was measured by absorbance values. LC50 was obtained by the logit method of the drug inhibition concentration calculation software (version 1.1.0).

### 2.13 Luciferase reporter assay

The binding sites of BRD4 and miR‐377 were predicted by the online Target Scan software, and the mutant sequences and wild sequences of the BRD4 and miR‐377 binding sites were designed according to the predicted results. The BRD4 mutant sequence and the wild sequence fragment were cloned and bound to the PGL‐3 vector, and the 293T cells were cotransfected with the miR‐377 mimics or miR‐377 negative control, respectively. In addition, wild‐type sequences were conjugated to miR‐377 mimics or miR‐377 negative controls, respectively. Based on the transfection, the samples were grouped into MT+mimics group, MT+NC group, WT+mimics group and WT+NC group. After 48 hours of transfection, the fluorescence activity intensity of each group was measured using a luciferase kit (purchased from Beijing Yuanping Biotechnology Co., Ltd.).

### 2.14 Statistical analysis

SPSS22.0 software was performed for statistical analysis. The Kaplan‐Meier method was used to draw survival curve. Pearson correlation analysis was calculated the relationship between lncRNA and miRNA. Student's *t* test and chi‐square test were processed to estimate the difference between two groups, while one‐way ANOVA was used to calculate the difference among more than three groups. The threshold of significance was *P* < .05.

## 3 RESULTS

### 3.1 LncRNA POU6F2‐AS2 was over‐expressed in colon cancer

**Figure 1:**
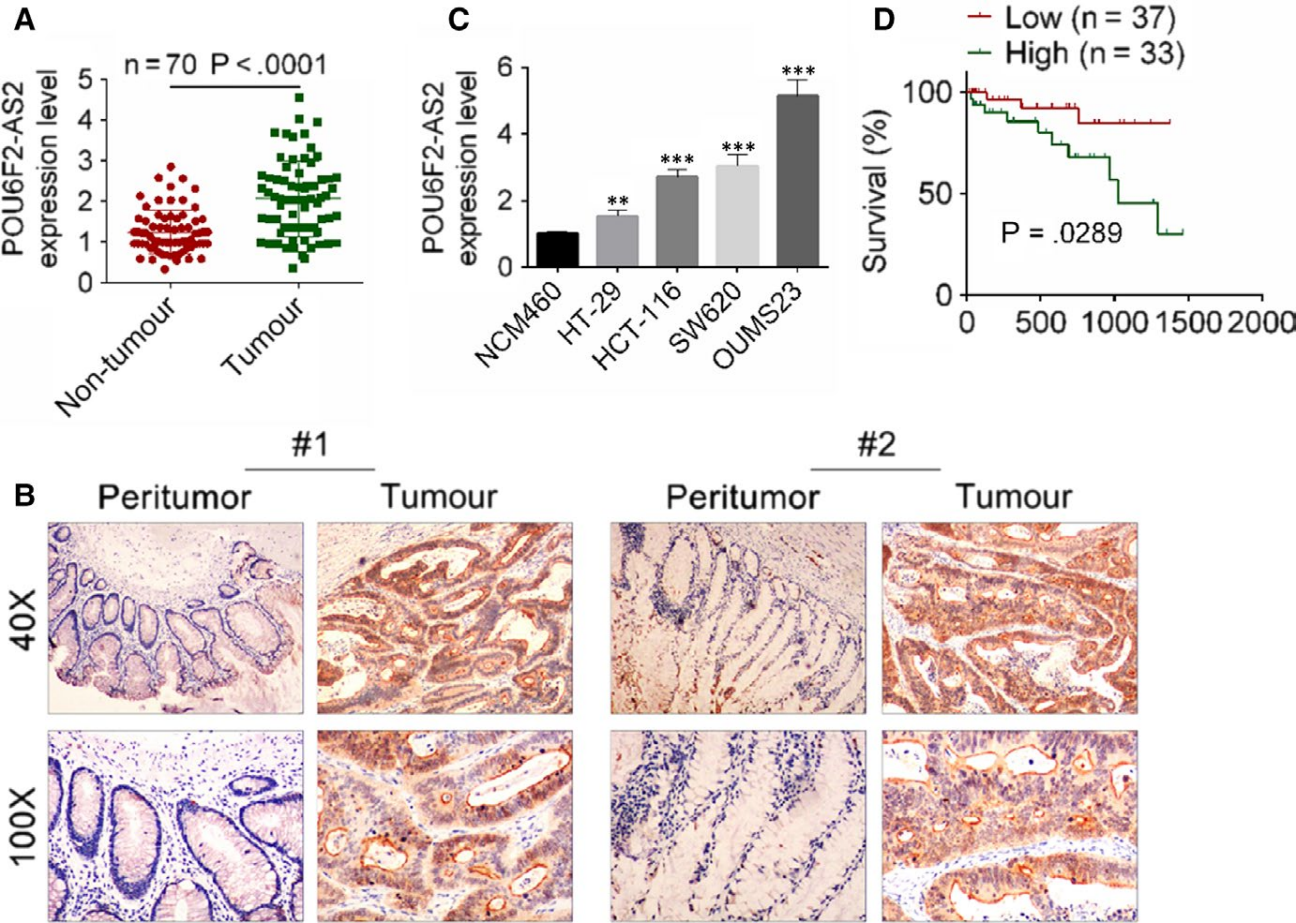
POU6F2‐AS2 expression level and related survival curve. A, POU6F2‐AS2 expression level in colon cancer tissue and adjacent normal tissues were detected by RT‐PCR, ^***^ *P* < .001. B, In situ hybridization for POU6F2‐AS2 in colon cancer tissue and adjacent normal tissues. C, POU6F2‐AS2 expression level in colon cancer cell lines (HT‐29, HCT‐116, SW620 and OUMS23) and non‐cancerous colon mucosal epithelial cell lines (NCM460) were detected by RT‐PCR. ^**^ *P* < .01 and ^***^ *P* < .001 vs NCM460. D, survival curve of colon cancer patients with low and high POU6F2‐AS2 expression level by Kaplan‐Meier survival analysis. Mean ± standard deviation was used to present the data

To explore the molecular mechanism of lncRNA POU6F2‐AS2 in colon cancer, the expression of lncRNA POU6F2‐AS2 was detected in colon cancer, and the relationship between the level of lncRNA POU6F2‐AS2 and survival time was also investigated. Association between clinicopathological features and lncRNA POU6F2‐AS2 expression in 70 colon patients were shown in Table 2. Based on the mean expression value of lncRNA POU6F2‐AS2, 70 patients were divided into low lncRNA POU6F2‐AS2 group (n = 37) and high lncRNA POU6F2‐AS2 group (n = 33). General clinical indexes, such as ages, gender, location, tumour size, differentiation and depth of invasion, were without significant difference between two groups. However, other clinicopathological features, including AJCC stage, vascular invasion, lymph node metastasis and distant metastasis, were significantly different between two groups. Based on the results of qRT‐PCR and ISH, the expression level of lncRNA POU6F2‐AS2 was significantly higher in colon cancer tissue than adjacent normal tissues (*P* < .0001, Figure 1A,B). Consistent with experiment of tissues, the expression of lncRNA POU6F2‐AS2 was significantly higher in HT‐29, HCT‐116, SW620 and OUMS23 cell lines than NCM460. Moreover, the lncRNA POU6F2‐AS2 expression was highest in OUMS23 cell lines (*P* < .001; Figure 1C). The lncRNA POU6F2‐AS2 expression in HT‐29 was lower than that in OUMS23 cell lines, but the value was significant higher than normal (*P* < .01). Similarly, the result of survival curve showed that patients with low lncRNA POU6F2‐AS2 expression always had longer survival time (*P* = .0289, Figure 1D).

**Table 2:** Association between clinicopathological features and POU6F2‐AS2 expression in 70 colon patients

Characteristics	Number of patients	POU6F2‐AS2 Low expression (<medin)	POU6F2‐AS2 High expression （≥medin)	*P* value
Number	70	37	33	
Ages(y)				
<60	39	22	17	.504
≥60	31	15	16	
Gender				
Female	38	18	20	.316
Male	32	19	13	
Location				
Left	30	15	15	.678
Right	40	22	18	
Tumour size				
≤3	35	21	14	.231
>3	35	16	19	
AJCC stage				
I	22	17	5	.019*
II	19	10	9	
III	17	7	10	
IV	12	3	9	
Differentiation				
Well	21	12	9	.258
Moderately	25	10	15	
Poorly	24	15	9	
Vascular invasion				
Yes	31	10	21	.002**
No	39	27	12	
Depth of invasion				
T_1_	17	12	5	.230
T_2_	17	10	7	
T_3_	18	7	11	
T_4_	18	8	10	
Lymph node metastasis				
N_0_	29	21	7	.005**
N_1_	20	10	10	
N_2_	21	6	15	
Distant metastasis				
M_0_	37	25	12	.009**
M_1_	33	12	21	

Note

The mean expression level of POU6F2‐AS2 was chosen as the threshold to divide patients into groups with low and high expression. Chi‐square test was used to estimate the difference of clinical features between two groups.

* *P* > .05.

** *P* < .01.

### 3.2 Overexpression of lncRNA POU6F2‐AS2 promoted proliferation and survival of colon cancer cells

**Figure 2:**
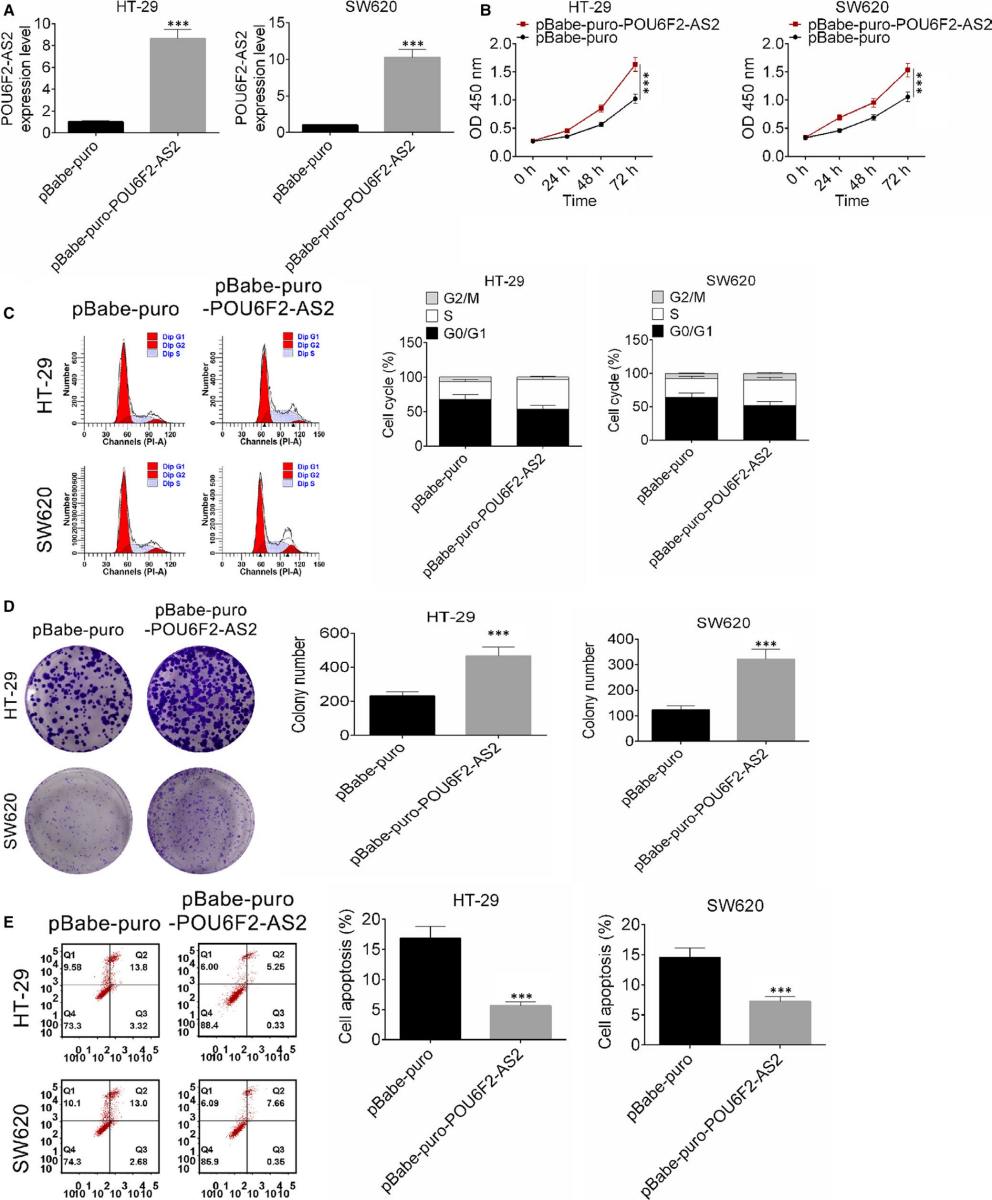
Overexpression of POU6F2‐AS2 promoted cell proliferation and cell cycle of colon cancer cells. A, The expression of POU6F2‐AS2 in HT‐29 and SW620 cell lines after transfected by pBabe‐puro‐POU6F2‐AS2 plasmid. B, The proliferation of HT‐29 and SW620 cell lines after transfected by pBabe‐puro‐POU6F2‐AS2 plasmid. C, Cell cycle of HT‐29 and SW620 cell lines after transfected by pBabe‐puro‐POU6F2‐AS2 plasmid. D, Clone number of HT‐29 and SW620 cell lines after transfected by pBabe‐puro‐POU6F2‐AS2 plasmid. E, The apoptosis of HT‐29 and SW620 cell lines after transfected by pBabe‐puro‐POU6F2‐AS2 plasmid. Mean ± standard deviation was used to present the data. ^***^ *P* < .001

After transfected by pBabe‐puro‐POU6F2‐AS2 plasmid, the expression of lncRNA POU6F2‐AS2 in HT‐29 and SW620 cell lines was significantly higher than control (Figure 2A, *P* < .001), indicating that the transfection was successful. Interestingly, up‐regulated lncRNA POU6F2‐AS2 significantly promoted the proliferation of colon cancer cells (Figure 2B, *P* < .001). In addition, after transfected by pBabe‐puro‐POU6F2‐AS2 plasmid, S phase of cell cycle was significantly increased (Figure 2C). Clone number of HT‐29 and SW620 cell lines after transfected by pBabe‐puro‐POU6F2‐AS2 plasmid was significant larger (D). Similarly, the number of apoptotic cells in both cell lines was larger, indicating that apoptosis was significantly improved by pBabe‐puro‐POU6F2‐AS2 (*P* < .001, Figure 2E). These results indicated that overexpression of lncRNA POU6F2‐AS2 promoted cell proliferation and cell cycle of colon cancer cells.

### 3.3 Down‐regulation of lncRNA POU6F2‐AS2 inhibited cell proliferation and induced cell cycle arrest of colon cancer cells

**Figure 3:**
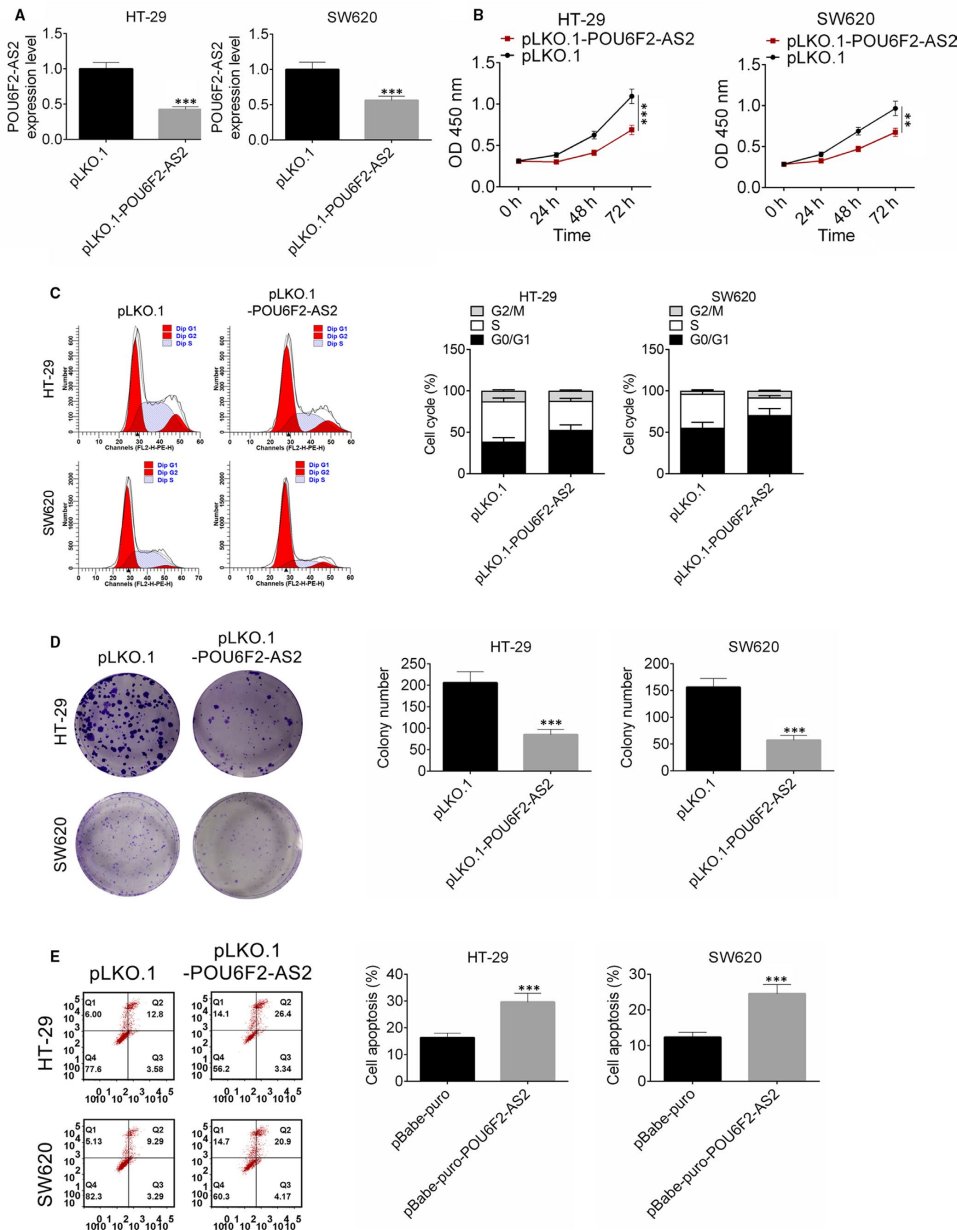
Down‐regulation of POU6F2‐AS2 inhibited cell proliferation and induced cell cycle arrest of colon cancer cells. A, The expression of POU6F2‐AS2 in HT‐29 and SW620 cell lines after transfected by pLKO.1‐POU6F2‐AS2 plasmid; B, the proliferation of HT‐29 and SW620 cell lines after transfected by pLKO.1‐POU6F2‐AS2 plasmid; C, cell cycle of HT‐29 and SW620 cell lines after transfected by pLKO.1‐POU6F2‐AS2 plasmid; D, clone number of HT‐29 and SW620 cell lines after transfected by pLKO.1‐POU6F2‐AS2 plasmid; E, the apoptosis of HT‐29 and SW620 cell lines after transfected by pLKO.1‐POU6F2‐AS2 plasmid. Mean ± standard deviation was used to present the data. ^***^ *P* < .001

After transfected by pLKO.1‐POU6F2‐AS2 plasmid, the expression of lncRNA POU6F2‐AS2 in HT‐29 and SW620 cell lines was significantly lower than control (Figure 3A, *P* < .001), indicating that the transfection was successful. Interestingly, down‐regulated of lncRNA POU6F2‐AS2 significantly inhibited the proliferation of colon cancer cells (Figure 3B, *P* < .001). In addition, after transfected by pLKO.1‐POU6F2‐AS2 plasmid, cell cycle of HT‐29 and SW620 cells was arrested (Figure 3C). Similarly, the colony number of in both cell lines was fewer, indicating that colony formations were significantly inhibited by pLKO.1‐POU6F2‐AS2 (*P* < .001, Figure 3D). Besides, increase in cell apoptosis was observed in pBabe‐puro‐POU6F2‐AS2 in HT‐29 and SW620 cell lines (*P* < .001, Figure 3E). These results indicated that silencing of lncRNA POU6F2‐AS2 inhibited cell proliferation and induced cell cycle arrest of colon cancer cells.

### 3.4 Down‐expression of lncRNA POU6F2‐AS2 leads to 5‐FU insensitivity

**Figure 4:**
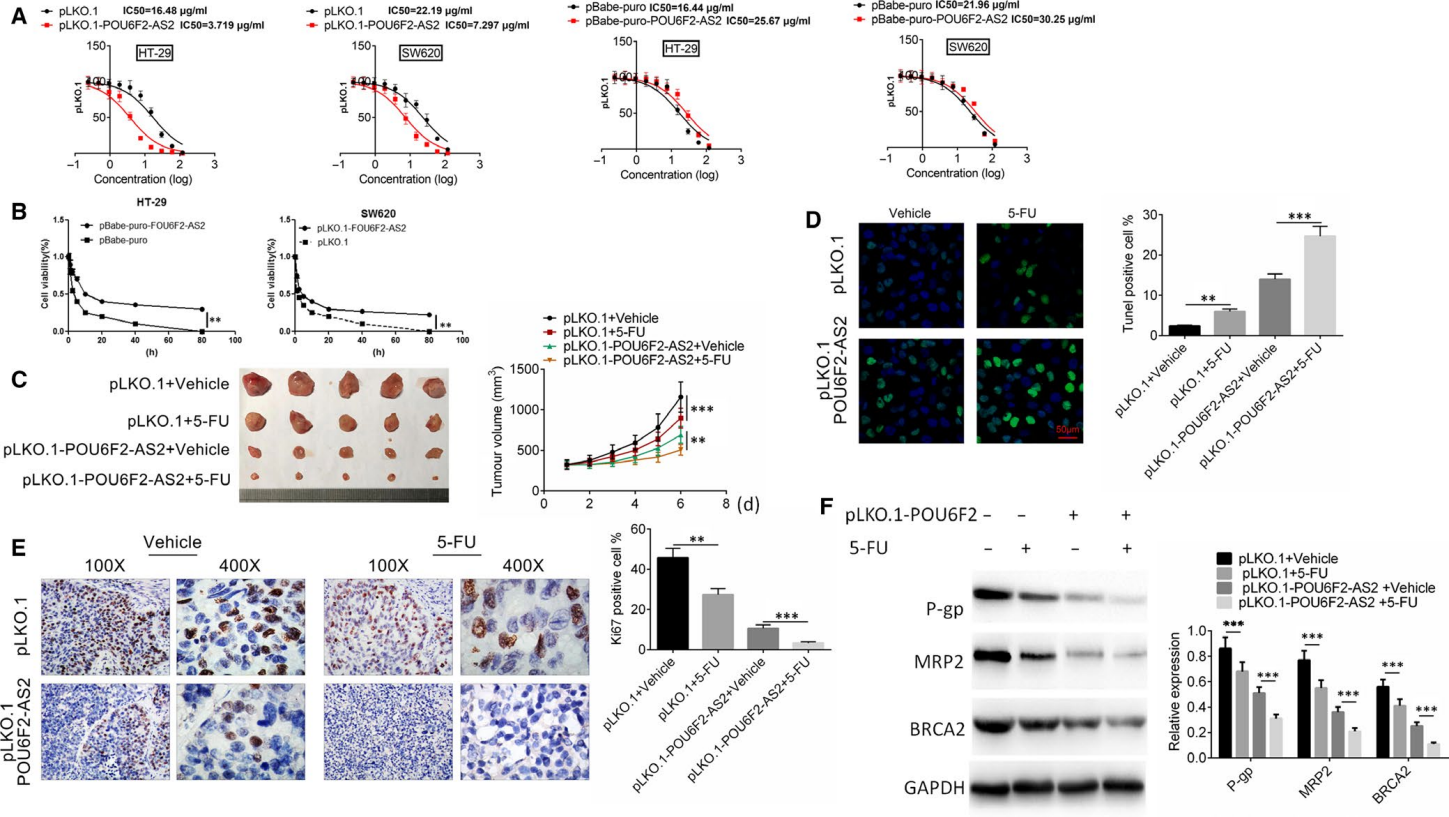
Down‐expression of POU6F2‐AS2 leads to 5‐FU insensitivity. A, LC50 assay. B, Cell viability by CCK8 assay after transfected by pBabe‐puro‐POU6F2‐AS2 plasmid or pLKO.1‐POU6F2‐AS2 plasmid. C, Tumour volume after transfection and drug treatment in four groups (pLKO.1 + Vehicle group, pLKO.1 + 5‐FU group, pLKO.1‐POU6F2‐AS2 + Vehicle group and pLKO.1‐POU6F2‐AS2 + 5‐FU group). D, The proportion of TUNEL‐positive cells in four groups. E, The proportion of Ki67‐positive cells in four groups. F, The protein levels of cancer resistance‐related gene (P‐gp, MRP2 and BRCA2). Mean ± standard deviation was used to present the data. ^**^ *P* < .01 and ^***^ *P* < .001

The LC50 of pLKO.1 and pBabe‐puro were 16.48 μg/mL, 16.44μg/mL (HT‐29), 22.19 μg/mL and 21.96 μg/mL (SW620), resepctively, while the LC50 of pLKO.1‐POU6F2‐AS2 and pBabe‐puro‐POU6F2‐AS2 was 3.719 μg/mL, 7.297 μg/mL (HT‐29), 25.67 μg/mL and 30.25 μg/mL (SW620), respectively (Figure 4A). After transfected by pBabe‐puro‐POU6F2‐AS2 plasmid, the cell viability was significantly decreased than pBabe control group. The opposite result was obtained after transfected by pLKO.1‐POU6F2‐AS2 plasmid (*P* < .01, Figure 4B).The results of vivo xenograft model showed that cell tumour formation was slower and tumour volume was smaller after lncRNA POU6F2‐AS2 was down‐regulated. At the 6th day after 5‐FU or vehicle treatment, the difference was statistically significant (*P* < .001, Figure 4C). After 5‐FU treatment, lncRNA POU6F2‐AS2 transfection or lncRNA POU6F2‐AS2 +5‐FU combined treatment could significantly increase the per cent of TUNEL‐positive cells and decrease the proportion of Ki67‐positive cells (*P* < .01, Figure 4D,E). 5‐Fu is converted into a fluorouracil dehydronucleotide in vivo, specifically binding to thymidine nucleotide synthase, affecting the synthesis of cellular DNA and ultimately leading to cell death. Interestingly, PLKO.1‐POU6F2 transfection significantly changed the expression of some cancer resistance‐related gene (P‐gp, MRP2 and BRCA2) in this study (*P* < .001, Figure 4F). The results showed that above treatment enhanced the apoptosis and reduced proliferation. More importantly, the changed level of apoptosis and proliferation in pLKO.1‐POU6F2‐AS2 +5‐FU treatment was larger than the sum of the other two groups, which indicating down‐regulated lncRNA POU6F2‐AS2‐enhanced sensitivity of 5‐FU by decreasing the expression of cancer resistance‐related gene.

### 3.5 LncRNA POU6F2‐AS2 combined with miR‐377

**Figure 5:**
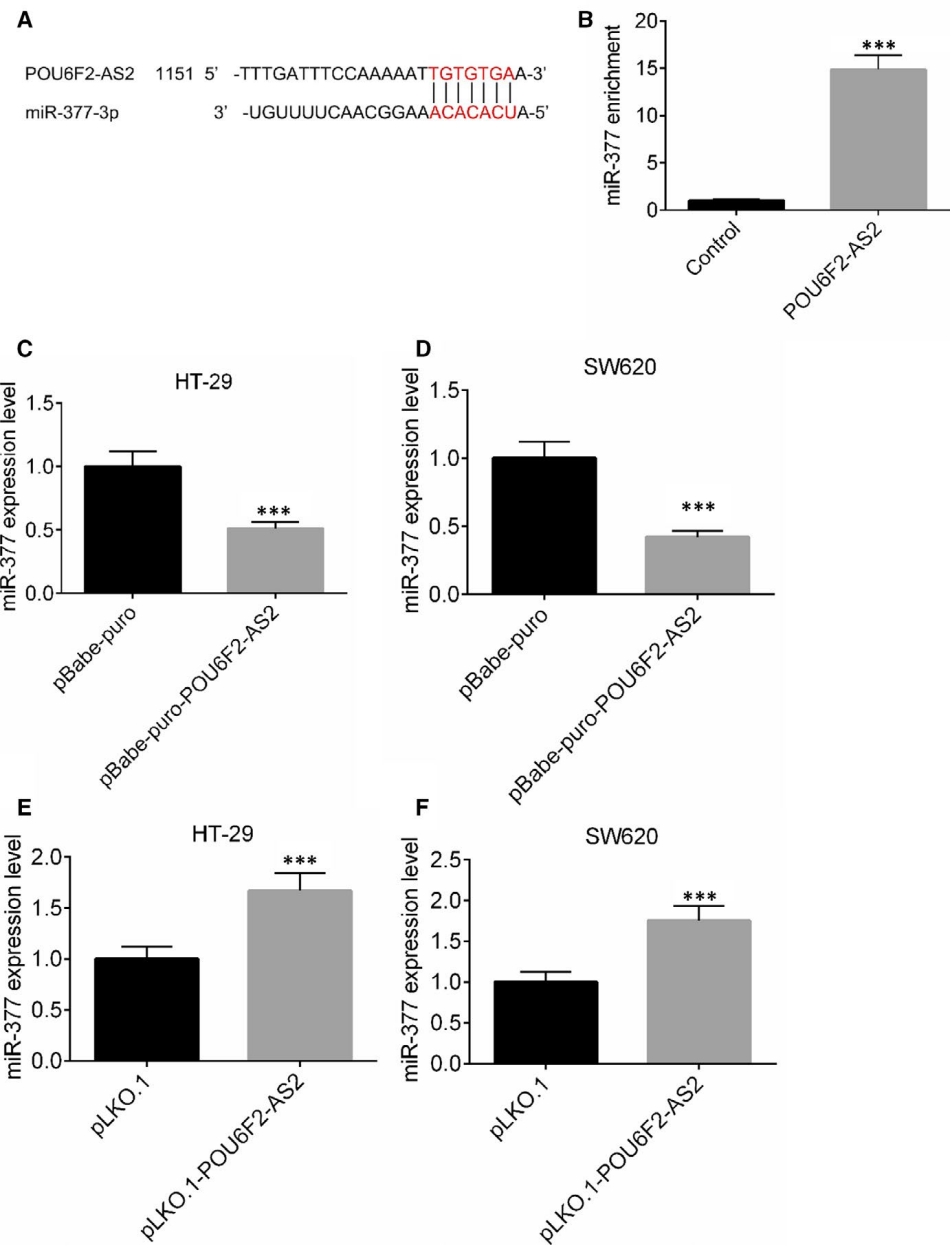
POU6F2‐AS2 combined with miR‐377. A, The binding site of POU6F2‐AS2 and miR‐377 predicated by catRAPID analysis. B, Pull‐down assay for the enrichment of miR‐377 in HT‐29 cell lines. C, miR‐377 expression level in HT‐29 after pBabe‐puro‐POU6F2‐AS2 plasmid transfected. D, miR‐377 expression level in SW620 after pBabe‐puro‐POU6F2‐AS2 plasmid transfected. E, miR‐377 expression level in HT‐29 after pLKO.1‐POU6F2‐AS2 plasmid transfected. F, miR‐377 expression level in SW620 after pLKO.1‐POU6F2‐AS2 plasmid transfected. Mean ± standard deviation was used to present the data. ^***^ *P* < .001

Based on the catRAPID analysis, the binding site of lncRNA POU6F2‐AS2 and miR‐377 was obtained and shown in Figure 5A. After lncRNA POU6F2‐AS2 in SW620 was up‐regulated, an RNA pull‐down experiment was performed. The substrate of pull‐down assay was used to detect the enrichment of miR‐377 by qRT‐PCR. After lncRNA POU6F2‐AS2 transfection, the miR‐377 enrichment ability miR‐377 was significantly higher than control. miR‐377 was enriched by lncRNA POU6F2‐AS2, indicating the presence of lncRNA POU6F2‐AS2 in combination with miR‐377 (*P* < .001, Figure 5B). In SW620 and HT‐29 cells lines, pBabe‐puro‐POU6F2‐AS2 could down‐regulate the expression of miR‐377, while pLKO.1‐POU6F2‐AS2 could increase the expression level of miR‐377 (*P* < .001, Figure 5C‐F).

### 3.6 The correlation among miR‐377, BRD4 and lncRNA POU6F2‐AS2

**Figure 6:**
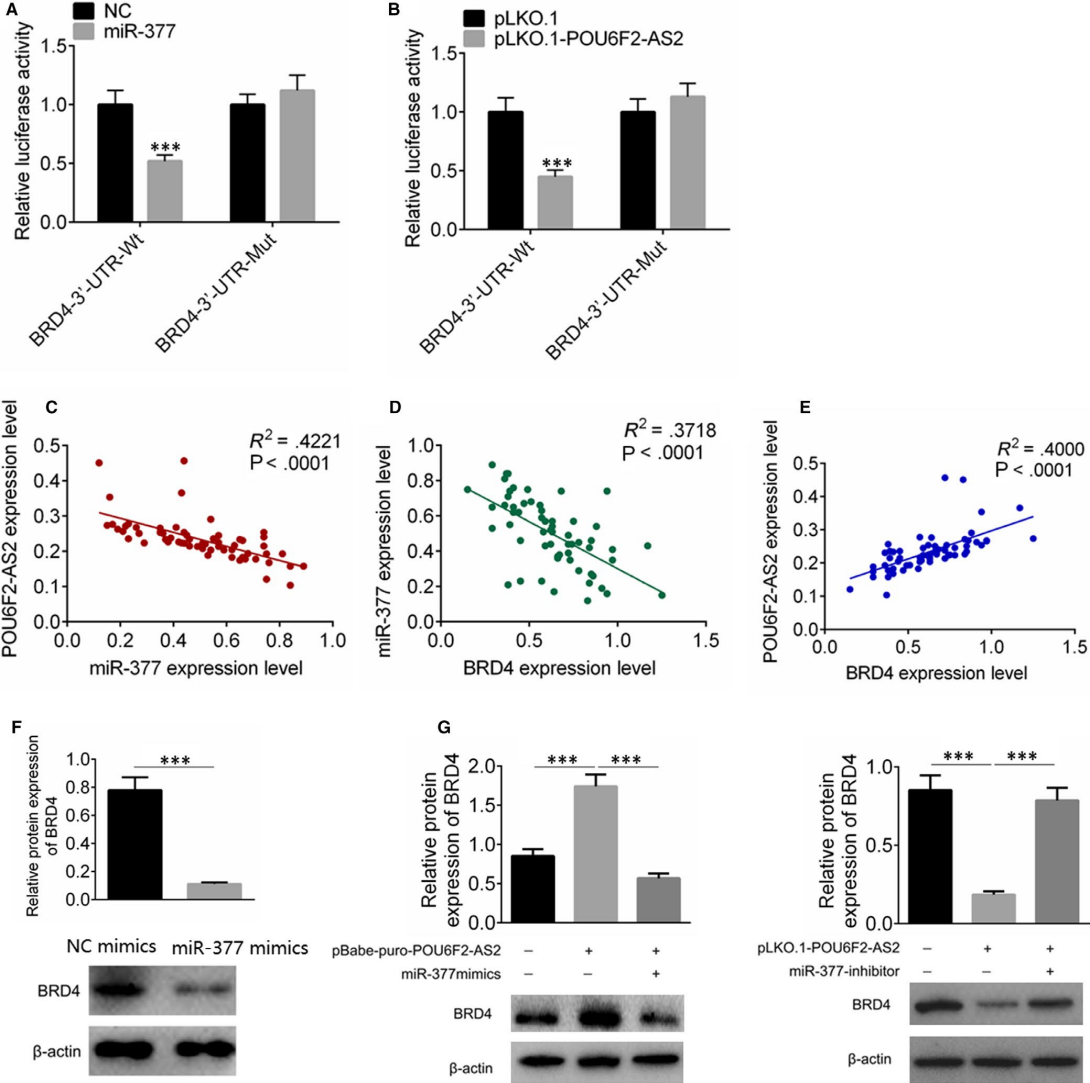
The fluorescence activity and the correlation among miR‐377, BRD4 and POU6F2‐AS2. A, The fluorescence activity after transfected by miR‐377 mimics; B, The fluorescence activity after transfected by pLKO.1‐POU6F2‐AS2 plasmid; C, D and E, The expression of POU6F2‐AS2 was negatively correlated with miR‐377, the expression of miR‐377 was negatively correlated with BRD4, and the expression of POU6F2‐AS2 was positively correlated with BRD4. Pearson correlation analysis was calculated the relationship between lncRNA and miRNA. F, G, The expression of BRD4 in colon cancer cells was examined by Western blot assay. ^***^ *P* < .001

After miR‐377 was up‐regulated, the fluorescence activity of miR‐377 + BRD4‐3′‐UTR group was decreased (*P* < .001, Figure 6A), while the fluorescence activity of miR‐377+ BRD4‐3′‐UTR mutation was with no significant difference compared with control (Figure 6B). The mRNA expression levels of lncRNA POU6F2‐AS2, miR‐377 and BRD4 were examined, and the correlation among the three factors was analysed. The expression of lncRNA POU6F2‐AS2 was negatively correlated with miR‐377, the expression of miR‐377 was negatively correlated with BRD4, and the expression of lncRNA POU6F2‐AS2 was positively correlated with BRD4 (*P* < .0001, Figure 6C‐E). The transfection of pBabe‐puro‐POU6F2‐AS2 increased the BRD4 expression, while miR‐377 mimics significantly decreased the expression of BDR4 in colon cancer cells (Figure 6F). Similarly, the transfection of pLKO.1‐POU6F2‐AS2 could significantly decrease the expression of BDR4, and miR‐377 inhibitor could rescue the effect (Figure 6G).

### 3.7 Rescue experiments

**Figure 7:**
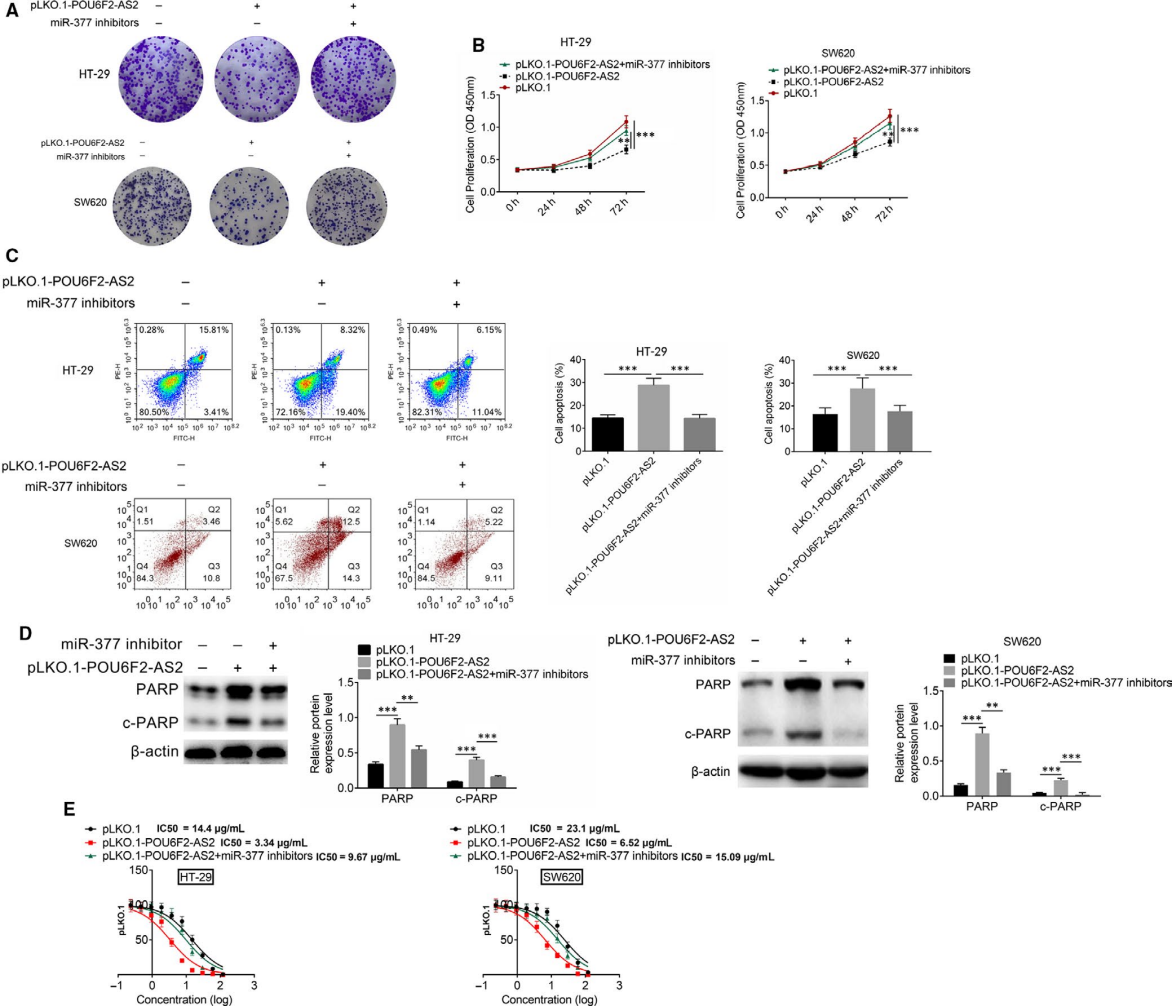
Rescue experiments. A, Clone number of HT‐29 and SW620 after transfected by pLKO.1‐POU6F2‐AS2 plasmid or miR‐377 inhibitors. B, Cell proliferation after transfected by pLKO.1‐POU6F2‐AS2 plasmid or miR‐377 inhibitors C, The proportion of apoptosis was detected after transfected by pLKO.1‐POU6F2‐AS2 plasmid or miR‐377 inhibitors. D, Western blot detected the expression levels of PARP and C‐PARP after transfected by pLKO.1‐POU6F2‐AS2 plasmid or miR‐377 inhibitors in 5‐FU treated cells. E, LC50 assay. Mean ± standard deviation was used to present the data. ^***^ *P* < .001

The rescue experiments contained three groups, including pLKO.1, pLKO.1‐POU6F2‐AS2 and pLKO.1‐POU6F2‐AS2 + miR‐377 inhibitors group. pLKO.1‐POU6F2‐AS2 was transfected into HT‐29 and SW620 cells, and miR‐377 inhibitors were transfected simultaneously, followed by CCK8 and crystal violet staining proliferation experiments. After pLKO.1‐POU6F2‐AS2 transfection, the number of cell clone was significantly fewer, while knocking down miR‐377 at the same time rescued the process (Figure 7A). In addition, pLKO.1‐POU6F2‐AS2 transfection could slow down the cell proliferation. Knocking down miR‐377 at the same time would decrease the inhibition of proliferation by pLKO.1‐POU6F2‐AS2 transfection (Figure 7B).After pLKO.1‐POU6F2‐AS2 transfection, the proportion of apoptosis increased. Knocking down miR‐377 at the same time would decrease the promotion of apoptosis (Figure 7C). The results of Western blot also showed that miR‐377 inhibitor rescued the affection of down‐regulated POU6F2‐AS2, and also recover the expression level of PARP and C‐PARP (Figure 7D). Similarly, in HT‐29 cell line, LC50 of pLKO.1, pLKO.1‐POU6F2‐AS2 and pLKO.1‐POU6F2‐AS2+miR377 inhibitors was 14.4 μg/mL, 3.34μg/mL and 9.67 μg/mL, respectively. While in SW620 cell line, LC50 of pLKO.1, pLKO.1‐POU6F2‐AS2 and pLKO.1‐POU6F2‐AS2+miR377 inhibitors was 23.1 μg/mL, 6.52 μg/mL and 15.09 μg/mL, respectively (Figure 7E).

## 4 DISCUSSION

In this study, lncRNA POU6F2‐AS2 was confirmed to be highly expressed in colon cancer, which was also associated with clinical pathology. Up‐regulated lncRNA POU6F2‐AS2 inhibited the expression of miR‐377, further up‐regulated the expression of BRD4 and ultimately promoted cell proliferation and cell cycle progression. In addition, high expression of lncRNA POU6F2‐AS2 led to 5‐FU insensitivity in colon cancer cells.

Previous studies have shown that lncRNA POU6F2‐AS2 can participate in DNA repair process. It was found to interact with Ybx1 protein and also participated in DNA damage response and regulation of cell survival.^20^ In gastric cancer, high expression of POU6F2‐AS2 is related to depth of tumour invasion and TNM stage.^21^ Based on lncRNA‐mRNA interactive network constructed by ping‐pang algorithm, Lv et al^11^ found that lncRNA POU6F2‐AS2 was associated with the occurrence and development of oesophageal cancer. Moreover, lncRNA POU6F2‐AS2 interacted with Ybx1 and further participated in development of oesophageal squamous cell carcinoma.^20^ In this study, lncRNA POU6F2‐AS2 was highly expressed in colon cancer and was also associated with clinical pathology. Thereby, lncRNA POU6F2‐AS2 may be considered as a potential target for colon cancer treatment.

Interestingly, we also found that up‐regulated lncRNA POU6F2‐AS2 was found to inhibit the expression of miR‐377 and further up‐regulate the expression of BRD4 in colon cancer. MiRNAs play critical roles in different cancer biological processes, such as cell proliferation and cell apoptosis. In malignant melanoma, the expression of miR‐377 was silenced, E2F and MAP3K7/NF‐κB signalling pathway were activated, and metastatic and tumorigenic potentials were promoted.^22^ Hiroshi et al^23^ processed gene expression microarray and Western blot analysis, and found that E2F pathway was down‐regulated in colon cancer cells. Besides, targeting NF‐κB signalling pathway for CRC therapy has confirmed to be an effective strategy for colon cancer treatment.^24^ In addition, miR‐377 was also confirmed to target specificity protein 1, and further inhibit invasion and proliferation of glioblastoma cells.^25^ The expression of CD133 and VEGF was inhibited by miR‐377, and further suppressed the progression of oesophageal cancer cells.^26^ As shown in previous study, compared with CD133−, the tumorigenic potential of CD133+ cells was increased, which was due to interact with neighbouring carcinoma‐related fibroblasts.^27^ Apart from this, blocking VEGF expression significantly inhibited angiogenesis in colon cancer cells.^28^ Therefore, lncRNA POU6F2‐AS2 promoted cell proliferation by inhibiting the expression of miR‐377. In this study, the target gene BRD4 was also researched. Consistent with our research, BRD4 has been confirmed to involve in cell proliferation and cycle progression,^16^ and BRD4 inhibitor significantly inhibited cell metastasis and growth of colon cancer.^29^ Consequently, lncRNA POU6F2‐AS2 promoted cell proliferation by regulating miR‐377/BRD4 gene. It should be noted that in the colony formation experiments, cell number was decreased by 50%‐80% with POU6F2‐AS2 knockdown. However, both cell cycle and cell apoptosis assays showed that cell number was reduced by 10%‐15% with POU6F2‐AS2 knockdown. Thus, we think there are other underlying mechanisms that lncRNA POU6F2‐AS2 is acting besides regulating miR‐377/BRD4, which is also part of our future research.

More importantly, overexpression of lncRNA POU6F2‐AS2 was confirmed to strengthen the drug resistance of 5‐FU in this study. Drug resistance of colon cancer cells was the key to the efficacy of chemotherapy for colon cancer, which might lead to chemotherapy failure. Studying and understanding the drug resistance mechanism of colon cancer cells were crucial for reversing and reducing drug resistance, and improving the effect of chemotherapy. Colon cancer cells were with broad resistance to anticancer drugs both in vitro and in vivo. The pattern of cross‐resistance to natural compounds, such as doxorubicin and the Vinca alkaloids, suggests a pattern of pleiotropic drug resistance. 5‐FU was the most widely used drug in the treatment of colon cancer, and resistance to 5‐Fu was the main reason for failure of chemotherapy.^30^ Though various miRNAs were demonstrated to regulate 5‐FU resistance in colon cancer cells,^31^ lncRNAs and related mechanisms were rarely researched. In this paper, we focused on the inhibition of lncRNA POU6F2‐AS2 for 5‐FU resistance and confirmed it in vivo. After lncRNA POU6F2‐AS2 knockdown, tumour shrinkage was observed, and the expression of cancer resistance‐related gene (P‐gp, MRP2 and BRCA2) was down‐regulated, indicating lncRNA POU6F2‐AS2 knockdown enhanced sensitivity of 5‐FU. In previous studies, enhanced expression of P‐gp was confirmed to participate in DNA repair progress, which was critical for cisplatin resistance in colon adenocarcinoma cell lines.^32^ Besides, ABC transporter MRP2 was higher expressed in various human cisplatin‐resistant cell lines.^33^ Based on Sakai et al,^34^ inherent mutations of BRCA2 might induce resistance to cisplatin in breast and ovarian cancers. All above genes were closely related to cancer resistance. Thereby, lncRNA POU6F2‐AS2 knockdown regulated the cancer resistance.

In conclusion, lncRNA POU6F2‐AS2 promoted cell proliferation and drug resistance in colon cancer by regulating miR‐377/BRD4 axes. It might be a new target for colon cancer treatment.

## CONFLICTS OF INTEREST

All authors declare that they have no conflicts of interest.

## AUTHOR CONTRIBUTION

GX, HZ and JX performed the experiments. YW, YZ and MZ designed the research study. JX analysed the data. DZ, HZ and JX wrote the manuscript. All authors reviewed and approved the manuscript.

## Data Availability

The data used in the current study are available from the corresponding author on reasonable request.
